# Gamma Knife Stereotactic Radiosurgery With a Reduced Peripheral Dose: Outcomes in Uveal Melanoma Patients

**DOI:** 10.1016/j.adro.2026.102009

**Published:** 2026-02-12

**Authors:** Lily Kollitz, Shubhendu Mishra, Ashley Gao, Guneet Sodhi, Peter H. Tang, Yoichi Watanabe, Andrew S. Venteicher, Dara Koozekanani, Clara Ferreira, Evidio Domingo-Musibay, Jianling Yuan, Margaret A. Reynolds, Lindsey Sloan, Kathryn E. Dusenbery

**Affiliations:** a University of Minnesota, Minneapolis, Minnesota; b Department of Radiation Oncology, University of Minnesota, Minneapolis, Minnesota; c Retina Consultants of Minnesota, Minneapolis, Minnesota; d Storm Eye Institute, Medical University of South Carolina, Charleston, South Carolina; e Department of Neurosurgery, University of Minnesota, Minneapolis, Minnesota; f Department of Ophthalmology and Visual Neurosciences, University of Minnesota, Minneapolis, Minnesota; g Allina Health, Minneapolis, Minnesota

## Abstract

**Purpose:**

Gamma Knife stereotactic radiosurgery (GK SRS) is an established alternative option for treating uveal melanoma in appropriate candidates. Despite proven efficacy, the optimal GK SRS dose remains uncertain. This study evaluated the safety and efficacy of a reduced-dose protocol.

**Methods and Materials:**

In this retrospective study, 24 adult patients with medium- or large-sized uveal melanoma unsuitable for plaque brachytherapy underwent GK SRS, with a median peripheral dose of 24 Gy. Local failure was defined according to the Collaborative Ocular Melanoma Study criteria. Kaplan-Meier methods were used to estimate local tumor control and vision outcomes.

**Results:**

In this cohort, 50% of patients had medium-sized tumors, and 50% had large-sized tumors. Local tumor control was 100% at 1 year and 95% at 2 years. There was no statistically significant difference in local control between medium and large tumors (*P* = .303); notably, all confirmed failures occurred in the medium-sized tumor group. The eye preservation rate was 83.3%. Regarding visual toxicity, 69.6% of patients maintained vision above the legal blindness threshold at 1 year.

**Conclusion:**

With favorable visual preservation, the 24 Gy dose yielded local tumor control comparable to that of previously published higher-dose protocols. Further investigations are warranted to define the dose that best balances tumor control and vision preservation in uveal melanoma.

## Introduction

Uveal melanoma (UM) is the most common primary malignancy of the adult eye.^1^ Treatments for UM include enucleation, proton beam therapy, photodynamic radiation therapy, and plaque brachytherapy.^1^, ^2^, ^3^ The Collaborative Ocular Melanoma Study (COMS) has established that ^125^I plaque brachytherapy is an effective treatment modality for UM that also permits eye preservation.^4^ Plaque brachytherapy involves suturing a radioactive plaque to the sclera, which delivers radiation therapy to the uveal lesion for 3 to 7 days before removal.^5^

Although plaque brachytherapy is the most widely used treatment for UM, alternative therapies, such as Gamma Knife stereotactic radiosurgery (GK SRS), have been employed in patients who are not ideal candidates for plaque brachytherapy or because of patient preference.^6^ Patients who are deemed poor candidates for plaque brachytherapy have peripapillary lesions or large melanomas that do not meet the criteria for effective plaque treatment.^7^ Since plaque brachytherapy is the standard of care for small- to medium-sized tumors, and GK SRS is often reserved for larger tumors,^7^ head-to-head comparisons of local control (LC) rates and side effects are difficult because of large differences in baseline patient characteristics. Nonetheless, a meta-analysis by Parker et al^6^ reported comparable c rates for plaque brachytherapy and GK SRS, despite patients in the GK SRS cohort having worse baseline prognostic features due to larger tumor volumes at presentation.

With the increasing clinical use of GK SRS, interest has grown in reducing the delivered radiation dose to limit adverse effects. In a study by Dinca et al,^8^ significant reductions in adverse effects were observed when comparing GK SRS doses of 50 to 70 Gy, 45 Gy, and 35 Gy, with no significant difference in 5-year survival among these groups. Additional studies are needed to determine which GK SRS dose for UM achieves LC while maintaining limited ocular toxicity. The present study aimed to assess the efficacy and safety outcomes in 24 patients who received a reduced peripheral GK SRS dose of approximately 24 Gy.

## Methods and Materials

### Subjects

This retrospective, noncomparative, institutional review board-approved study included 24 adult patients diagnosed with medium- or large-sized UM unsuitable for plaque brachytherapy who were treated with GK SRS between 2020 and 2023 at a single institution. Patients were excluded if they had extraocular involvement or metastases at the time of GK SRS. Of the 24 patients, 62.5% (15/24) were men and 37.5% (9/24) were women. The mean age at treatment was 69.5 years. The right eye was affected in 33.3% (8/24) of patients, and the left eye in 66.7% (16/24).

T-staging for included patients was limited by inconsistent use of diameter measurements and by a preference among treating providers for COMS classification over American Joint Committee on Cancer staging. Overall, 50% (12/24) of patients had insufficient clinical data to assign a T stage. Among the remaining patients, 20.8% (5/24) were stage T2a, 25.0% (6/24) were stage T3a, and 4.2% (1/24) were stage T4a. All patients (24/24) were N0 and M0 at the time of diagnosis. No patient had documented ciliary body involvement or extraocular extension; however, these features were not routinely documented in the available ophthalmology records.

### Efficacy and safety assessment

Treatment efficacy was defined as the LC failure rate following GK SRS. LC failure was defined using COMS Report No. 19 criteria: >15% increase in tumor height, >250 µm increase in basal diameter, extrascleral extension > 2 mm, or orbital recurrence confirmed on 2 exams or prompting salvage therapy/enucleation.^4^ Safety outcomes included visual acuity, cataract progression, and the incidence of radiation retinopathy, glaucoma, neovascularization, and enucleation.

### Examination and follow-up

All included patients underwent ophthalmologic evaluation before GK SRS. This assessment included best-corrected visual acuity (BCVA), slit-lamp examination, intraocular pressure (IOP) measurement, indirect ophthalmoscopy, color fundus photography, and ultrasonography to measure apical tumor height. Following this examination, patients received GK SRS.

Posttreatment evaluations were performed at 1, 3, 6, and 12 months, encompassing BCVA, IOP measurement, slit-lamp examination, and indirect ophthalmoscopy. At the 12-month visit, color fundus photography and ultrasonography were repeated. Screening for metastasis included chest radiographs, chest/abdomen/pelvis computed tomography, and liver function tests. All available survival and distant metastasis data were reviewed (follow-up range, 13-46 months; median, 28.5 months), and all subsequent ophthalmic data for patients (IOP measurements, BCVA, and apical thickness) after GK SRS were reported.

### Treatment technique

Gamma Plan software (Elekta Instruments) was used to generate magnetic resonance imaging-based dose plans for each patient, delivering radiation via a cobalt-60 source. Of the 24 patients, 21 received the prescribed peripheral dose of 24 Gy, 1 received 25 Gy, and 2 received 22 Gy. The isodose goal for the tumor periphery was set at 50% for 14 patients, 47% for 1 patient, and 54% to 65% for the remaining 9 patients. The maximum tumor dose was located at the 100% isodose line.

A neurosurgeon and a radiation oncologist collaborated on the planning process to minimize dose to critical structures (eg, the optic nerve and lens). A Leksell Gamma Knife Radiosurgery ICON system (Elekta Instruments) was employed for radiation delivery. Under general anesthesia, 2 immobilization precautions were used to ensure accurate dose targeting of the tumor: (1) suturing of extraocular muscles after supplemental retrobulbar anesthesia to restrict eye movement and (2) securing a Leksell G-frame to the patient’s skull after injecting supplemental local anesthesia. This allowed for a stable setup with negligible motion. Consequently, no additional safety margin (0 mm expansion) was added around the radiosurgical target delineated by the neurosurgeon and radiation oncologist.

Gamma Plan software was used to calculate the administered prescription dose, conformity index (100% isodose line/tumor volume), dose gradient index (50%/100% isodose line), tumor volume, and doses to critical structures. Patients were placed supine in a magnetic resonance imaging scanner, and GK SRS was then delivered in a single fraction.

### Endpoints and statistical analysis

All time-to-event analyses were performed with Python (v3.10) in Google Colab, using the lifelines and matplotlib libraries. Kaplan-Meier (KM) methods were used to estimate time-to-event outcomes, and comparisons between the medium and large tumor subgroups were evaluated using the log-rank test (2-sided). A *P* value < .05 was considered statistically significant. Time was measured from the date of radiosurgery to the first event or the last follow-up. Patients without an event were censored at their most recent follow-up date.

The following endpoints were defined:

• LC: time to local failure, defined according to COMS criteria.
• Metastasis-free survival: time to the development of distant metastatic disease.
• Enucleation-free survival: time to surgical removal of the treated eye.
• Blindness-free survival: time to BCVA ≤ 20/200 (legal definition of blindness per 20 Code of Federal Regulations 404.1581).
• Overall survival: time to death from any cause.

All figures display survival probability over time (in months) with accompanying at-risk tables. Analyses were conducted using an open-source, reproducible Python pipeline in Google Colab. Descriptive statistics, such as those for demographic and Gamma Knife parameters, were calculated in Microsoft Excel (Microsoft Corporation).

## Results

Of the 24 patients, 50% (12/24) had medium-sized tumors, and 50% (12/24) had large-sized tumors, per the COMS classification (Table 1). Tumor basal diameters were recorded when available, consistent with American Joint Committee on Cancer T-staging data. Among these patients, 37.5% were women, and 62.5% were men, with a mean age at treatment of 69.5 years. The right eye was affected in 33.3% (8/24) of patients, whereas 66.7% (16/24) had tumors in the left eye. None had lymph node involvement or metastasis at the time of GK SRS initiation.

**Table 1:** Demographic data of the study population

Category	Subcategory	n (%)
Sex	Women	9 (37.5)
	Men	15 (62.5)
Age	<50	1 (4.2)
	50-59	5 (20.8)
	60-69	6 (25.0)
	70-79	9 (37.5)
	80-89	3 (12.5)
Tumor size (COMS classification)	Medium	12 (50.0)
	Large	12 (50.0)
Laterality	Right	8 (33.3)
	Left	16 (66.7)
AJCC N-stage (lymph node involvement pre-GK SRS)	N0	24 (100)
	N1	0 (0)
AJCC M-stage (metastasis pre-GK SRS)	M0	24 (100)
	M1	0 (0)
AJCC T-stage (tumor pre-GK SRS)	Tx	12 (50)
	T2a	5 (20.8)
	T3a	6 (25)
	T4a	1 (4.2)

This retrospective study included 24 patients who underwent GK SRS, with distributions of age, sex, tumor size (per COMS criteria), laterality, and AJCC for N-, M-, and T-staging shown above.

*Abbreviations:* AJCC = American Joint Committee on Cancer; COMS = Collaborative Ocular Melanoma Study; GK SRS = Gamma Knife stereotactic radiosurgery.

Table 2 details the prescribed isodose lines (47%-65%) and peripheral doses (22-25 Gy). The median peripheral dose was 24 Gy, and the median isodose line was 50%. The average target tumor volume was 0.83 cm³ (SD, 0.46), with an average coverage of 98.92%. The mean dose gradient index was 2.97 (SD, 0.24), while the mean conformity index was 1.56 (SD, 0.27). The average maximum lens dose was 12.11 Gy (SD, 9.75), and the average maximum optic nerve dose was 10.13 Gy (SD, 5.08).

**Table 2:** Gamma Knife stereotactic radiosurgery treatment parameters

Parameters	Values
Prescribed isodose line (%), median (range)	50 (47-65)
Prescribed peripheral dose (Gy), median (range)	24 (22-25)
Target tumor volume (cm³), mean ± SD	0.83 ± 0.46
Treatment coverage of target volume (%), mean	98.92
Gradient index, mean ± SD	2.97 ± 0.24
Conformity index, mean ± SD	1.56 ± 0.27
Maximum dose to lens (Gy), mean ± SD	12.11 ± 9.75
Maximum dose to optic nerve (Gy), mean ± SD	10.13 ± 5.08

The median (mdn) and ranges of the prescribed isodose line and peripheral dose are 50% (47%-65%) and 24 Gy (22-25 Gy) respectively. The mean (μ) and standard deviation (SD) are shown for target tumor volume (0.83 cm³ ± 0.46 cm³), gradient index (2.97 ± 0.24), conformity index (1.56 ± 0.27), maximum dose to lens (12.11 Gy ± 9.75 Gy) and maximum dose to optic nerve (10.13 Gy ± 5.08 Gy). The mean (μ) treatment coverage of the target volume was 98.92%.

**Figure 1:**
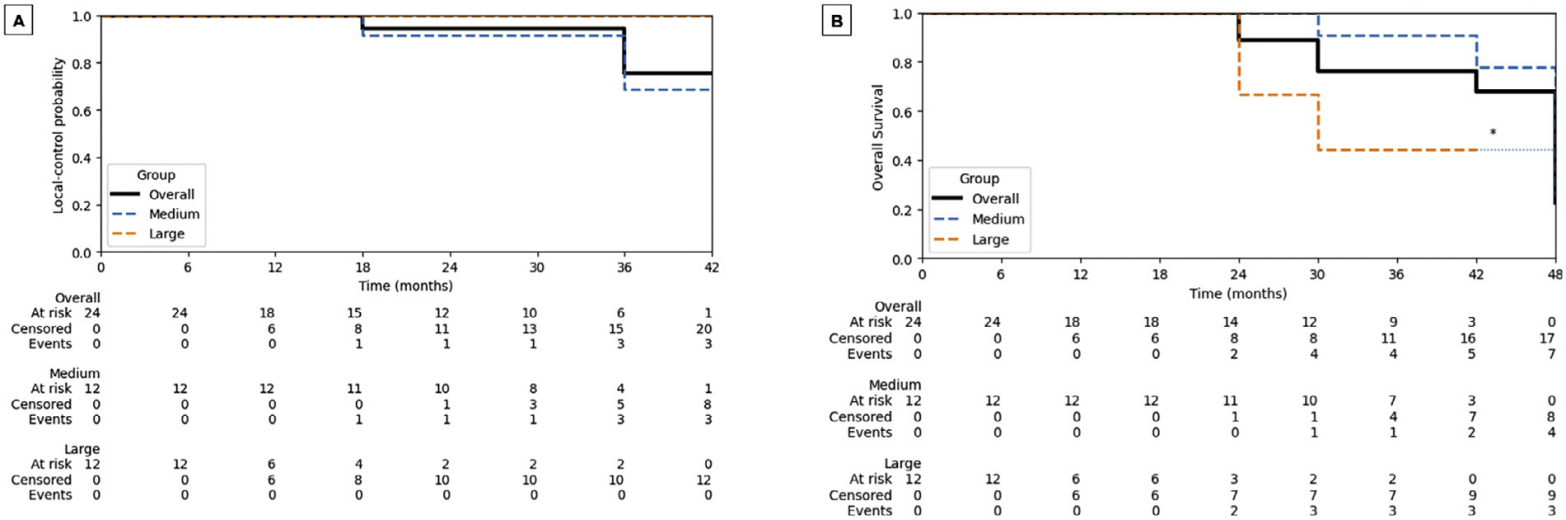
Kaplan-Meier curves for (A) local control and (B) overall survival of the study cohort, stratified by tumor size (medium vs large per the Collaborative Ocular Melanoma Study). Tables beneath each panel show the numbers of patients at risk, censored, and with events at each time point.

**Figure 2:**
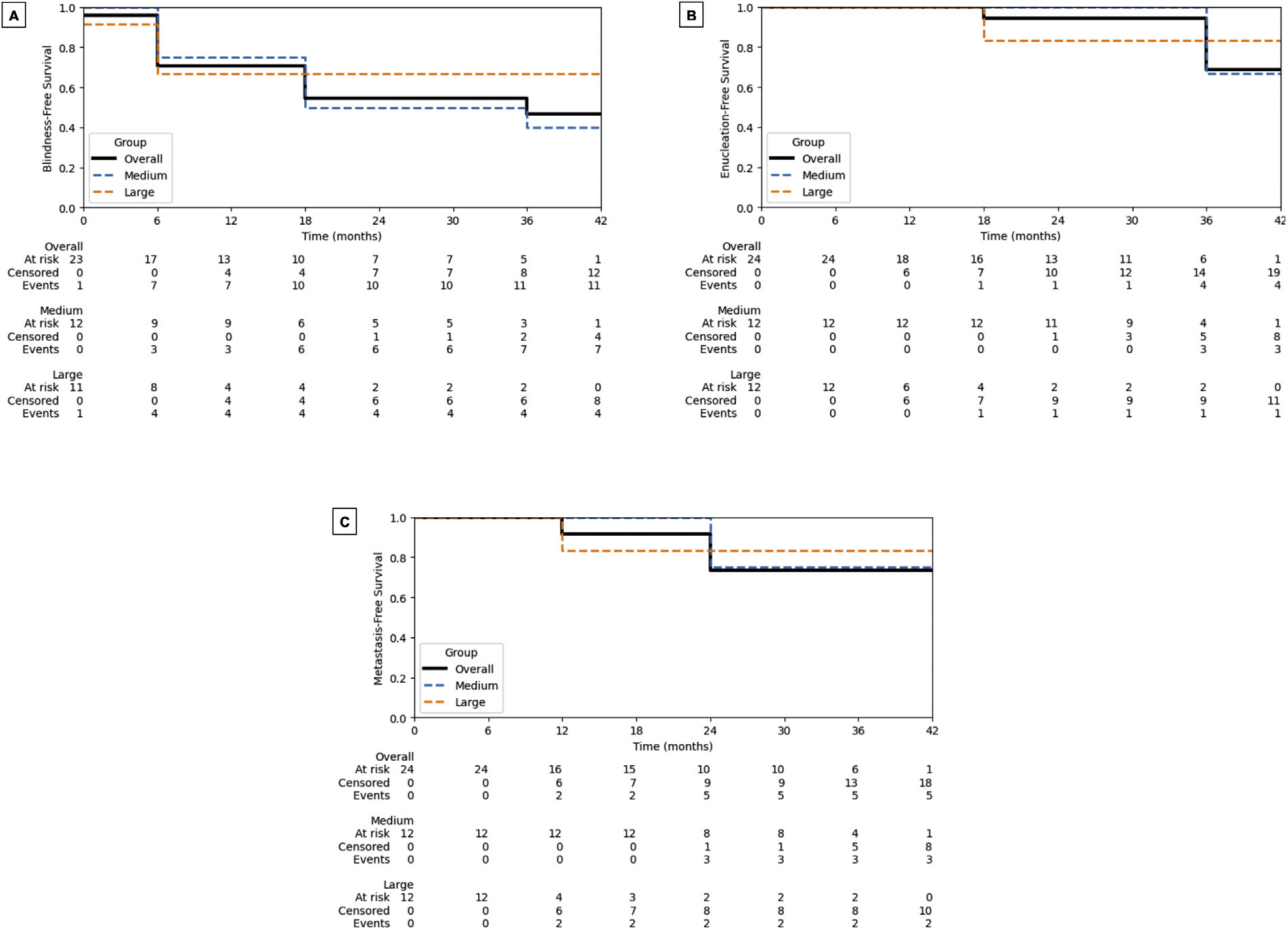
Kaplan-Meier curves for (A) blindness-free survival, (B) enucleation-free survival, and (C) metastasis-free survival in the study cohort, stratified by tumor size (medium vs large per the Collaborative Ocular Melanoma Study). Blindness-free survival was defined as best-corrected visual acuity ≤20/200. Tables beneath each panel show the numbers of patients at risk, censored, and with events at each time point.

Figure 1, Figure 2 display the KM curves for all endpoints. At 1 year, 100% (24/24) of patients met the COMS requirements for LC. The 1- and 2-year LC probabilities were 100% and 95%, respectively, for the overall cohort. Among the subgroups, medium tumors demonstrated 1- and 2-year LC rates of 100% and 91%, respectively, whereas large tumors maintained 100% LC throughout follow-up.

At the most recent follow-up, 12.5% (3/24) of patients did not meet the COMS requirements for LC and were therefore considered LC failures. Notably, all 3 failures occurred in the medium tumor size group. The remaining 87.5% (21/24) of patients had LC at their most recent follow-up appointment. There was no statistically significant difference in LC between medium and large tumors (log-rank *P* = .303). Additionally, 16.7% (4/24) of patients had transient tumor growth that did not meet criteria for local failure due to subsequent exams demonstrating tumor regression. In the KM analysis, 2 deaths occurred at 2 years and 4 deaths at 3 years; overall, 7 of 24 patients died during follow-up (Fig. 1B), with a log-rank *P* = .077 for overall survival (medium vs large).

Regarding visual outcomes, there was a statistically significant change in visual acuity at 1-year post-GK SRS, but no significant change in IOP. The mean logMAR (Logarithm of Minimal Angle of Resolution) BCVA worsened from 0.301 to 0.923, corresponding to a shift from approximately 20/40 to 20/170 Snellen equivalents, which approximates a 76% relative reduction in mean visual acuity. Prior to GK SRS, 23 of 24 patients had better than 20/200 vision; at 1-year post-GK SRS, 7 of these patients had visual acuity worse than 20/200, resulting in 69.6% retaining vision above the legal blindness threshold (see Fig. 2A for the blindness-free survival curve).

Regarding safety outcomes (Table 3), 45.8% of patients developed radiation retinopathy, 42.1% experienced cataract progression, and 8.3% reported dry eye symptoms. Neovascularization occurred in 12.5% of patients without subsequent enucleation and in 16.7% (4/24) of patients with subsequent enucleation (Fig. 2B). Distant metastases developed in 20.8% (5/24) of patients after GK SRS (Fig. 2C). Glaucoma and other ocular toxicities are summarized in Table 3.

**Table 3:** Gamma Knife stereotactic radiosurgery treatment complications

Category	n (%)
Radiation retinopathy	11 (45.8)
Cataract progression (of the 19 patients w/o prior cataract surgery)	8 (42.1)
Dry eye	2 (8.3)
Neovascularization	
Without subsequent enucleation	3 (12.5)
With subsequent enucleation	4 (16.7)
Distant metastases	5 (20.8)

Shown in this table are the counts and percentages (n [%]) of patients with treatment complications following Gamma Knife stereotactic radiosurgery. Complications included radiation retinopathy, cataract progression, dry eye, neovascularization (with and without subsequent enucleation), and distant metastasis.

**Figure 3:**
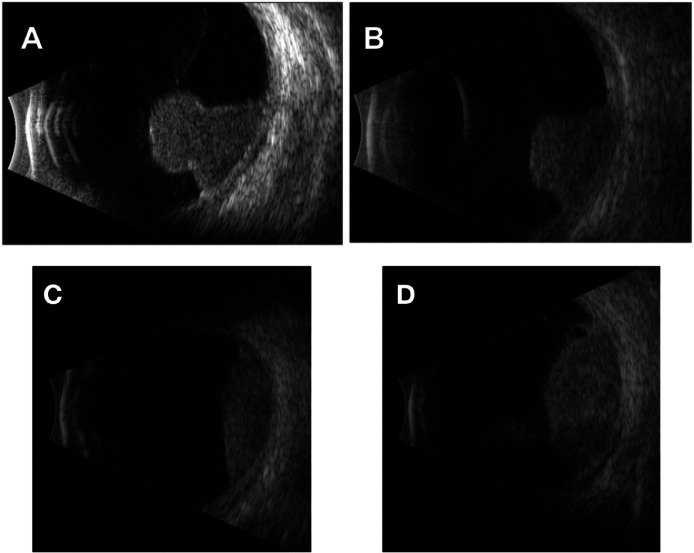
(A, B) Patient with local tumor control. (A) B-scan ultrasound of uveal melanoma before Gamma Knife stereotactic radiosurgery (GK SRS). (B) B-scan ultrasound of uveal melanoma post-GK SRS. (C, D) Patient with local tumor control failure. (C) B-scan ultrasound of uveal melanoma pre-GK SRS. (D) B-scan ultrasound of uveal melanoma post-GK SRS.

Figure 3A, B shows corresponding B-scan ultrasound and fundus photographs pre- and post-GK SRS for an example patient with successful treatment, and Fig. 3C, D shows those for a patient with LC failure.

## Discussion

The primary objective of this study was to evaluate the efficacy and safety of GK SRS for UM with a reduced peripheral dose of approximately 24 Gy. The results demonstrate that this reduced-dose protocol achieves LC rates comparable to those reported in larger meta-analyses and prior retrospective series using higher radiation doses.^6^^,^^9^^,^^10^ Specifically, Parker et al^6^ reported a 1-year LC rate of 96% in a meta-analysis with a median dose of 32 Gy. This aligns with the 1- and 2-year LC probabilities of 100% and 95%, respectively, observed in the present cohort. These findings support the hypothesis that doses at the lower end of the National Comprehensive Cancer Network-recommended range (18-45 Gy) are sufficient for LC in appropriately selected patients.

Recent time-dose-response modeling supports the efficacy of this dose range. Ehret et al^11^ demonstrated a dose-dependent effect of GK SRS for UM, predicting 2-year LC rates of >90% at 20 Gy and 95% at 22 Gy. Our protocol, using a median peripheral dose of 24 Gy, falls well within this effective therapeutic window. Furthermore, our data suggest that large tumor volume does not preclude effective control at this dose level. Although increased pretreatment UM apical thickness is a known predictor of recurrence,^12^ and data for very large tumors are limited,^9^^,^^13^^,^^14^ in our cohort, 50% of the tumors were classified as large (including 2 with apical thickness > 15 mm), and the large tumor subgroup achieved 100% LC throughout the follow-up period. Conversely, all 3 documented local failures occurred in the medium-sized tumor group.

Another notable finding in this series was the identification of transient tumor expansion. Four patients (16.7%) exhibited a temporary increase in tumor dimensions that subsequently regressed without intervention, likely explained by an inflammatory response of tumor cells to radiation, resulting in a brief, artificial enlargement. This phenomenon highlights the importance of distinguishing between pseudoprogression and true local failure to avoid unnecessary salvage enucleation. When applying the COMS criteria for local failure, which require confirmed growth or the need for salvage therapy, the LC rate remains high.

The LC outcomes in this study were achieved without the addition of a planning target volume margin (0 mm expansion). This approach relies on rigid immobilization to minimize intrafraction motion. As detailed in our methods, all patients were treated using retrobulbar anesthesia to induce extraocular muscle akinesia, combined with a Leksell G-frame for skull fixation. While this technique resulted in effective stabilization in this series, margin practices vary across institutions. Some centers advocate for margins of 1 to 1.5 mm to account for microscopic disease extension or setup uncertainty.^15^ Although the failures in this data set were not attributable to marginal misses, our institution has since adopted a pragmatic 1 mm margin expansion as the current practice. This change reflects institutional preference and should not be interpreted as a prescriptive recommendation, as margin strategies are inherently center-dependent.

The reduced-dose strategy achieved higher rates of vision preservation than those reported in higher-dose cohorts. Modorati et al^16^ reported that in patients receiving 30 to 50 Gy, median visual acuity declined to levels of legal blindness, representing a 94% relative reduction. In our study, 69.6% of patients retained vision better than 20/200 at 1 year, with a mean relative reduction in visual acuity of 76%. This suggests that constraining the peripheral dose to 24 Gy may result in less vision loss.

This study is limited by its retrospective design and small sample size, which restricts the statistical power of subgroup analyses. Additionally, T-staging was constrained by inconsistent documentation of basal diameters in the referring ophthalmology records and by incomplete documentation of ciliary body involvement and extraocular extension. However, with a median follow-up of 28.5 months and the use of standardized COMS definitions of failure, this study suggests that single-fraction GK SRS at a lower dose of 24 Gy offers LC rates comparable to those reported in current meta-analyses and recent time-dose-response modeling, while achieving minimal ocular morbidity. Larger, prospective studies are warranted to further define the optimal dose-de-escalation parameters and margin strategies for UM treated with GK SRS.

## Conclusions

This study demonstrates that single-fraction GK SRS with a reduced peripheral dose of 24 Gy provides durable LC comparable to outcomes reported in prior studies using higher doses, while offering a favorable toxicity profile for visual preservation. Notably, favorable control was achieved even in large tumors. Future multi-institutional studies with larger cohorts are needed to validate these findings and further refine the optimal dose and margin parameters for the treatment of UM.

## Disclosures

Lindsey Sloan receives research and travel support from GT Medical Technologies. Guneet Sodhi receives financial payment from Genentech Inc. Peter Tang receives financial payment from Genentech Inc and AbbVie/Regenx-Bio.

## References

[1] Wespiser M., Neidhardt E., Negrier S. (2023). Uveal melanoma: In the era of new treatments. Cancer Treat Rev.

[2] Blasi M.A., Pagliara M.M., Lanza A. (2018). Photodynamic therapy in ocular oncology. Biomedicines.

[3] Miao Y., Zheng T., Zhang Q. (2025). Efficacy and safety of proton radiotherapy in treating choroidal melanoma: A systematic review and meta-analysis. Radiat Oncol.

[4] Jampol L.M., Moy C.S., Murray T.G. (2002). The COMS randomized trial of iodine 125 brachytherapy for choroidal melanoma: IV. Local treatment failure and enucleation in the first 5 years after brachytherapy. COMS report no. 19. Ophthalmology.

[5] (1993). Design and methods of a clinical trial for a rare condition: The Collaborative Ocular Melanoma Study. COMS Report No. 3. Control Clin Trials.

[6] Parker T., Rigney G., Kallos J. (2020). Gamma knife radiosurgery for uveal melanomas and metastases: A systematic review and meta-analysis. Lancet Oncol.

[7] Yang J., Manson D.K., Marr B.P., Carvajal RD. (2018). Treatment of uveal melanoma: Where are we now?. Ther Adv Med Oncol.

[8] Dinca E.B., Yianni J., Rowe J. (2012). Survival and complications following γ knife radiosurgery or enucleation for ocular melanoma: A 20-year experience. Acta Neurochir (Wien).

[9] Sarici A.M., Pazarli H. (2013). Gamma-knife-based stereotactic radiosurgery for medium- and large-sized posterior uveal melanoma. Graefes Arch Clin Exp Ophthalmol.

[10] Schirmer C.M., Chan M., Mignano J. (2009). Dose de-escalation with gamma knife radiosurgery in the treatment of choroidal melanoma. Int J Radiat Oncol Biol Phys.

[11] Ehret F., Fürweger C., Liegl R. (2024). Tumor control probability and time-dose-response modeling for stereotactic radiosurgery of uveal melanoma. Int J Radiat Oncol Biol Phys.

[12] Kal Omar R., Hagström A., Dahlander S., Carlsson Tedgren Å, Stålhammar G. (2022). A prognostic score for the prediction of local treatment failure in plaque brachytherapy of uveal melanoma. Adv Radiat Oncol.

[13] Reynolds M.M., Whitaker T.J., Parney I.F. (2016). Carbon fiducials for large choroidal melanoma treated with gamma knife radiosurgery. Acta Ophthalmol.

[14] Arnett A.L.H., Reynolds M.M., Pulido J.S., Parney I.F., Laack NN. (2017). Gamma Knife stereotactic radiosurgery for the treatment of primary and metastatic ocular malignancies. Stereotact Funct Neurosurg.

[15] Haas A., Pinter O., Papaefthymiou G. (2002). Incidence of radiation retinopathy after high-dosage single-fraction gamma knife radiosurgery for choroidal melanoma. Ophthalmology.

[16] Modorati G., Miserocchi E., Galli L., Picozzi P., Rama P. (2009). Gamma knife radiosurgery for uveal melanoma: 12 years of experience. Br J Ophthalmol.

